# Circadian reprogramming of inflammation and metabolism in chronic kidney disease

**DOI:** 10.1080/0886022X.2026.2718935

**Published:** 2026-09-06

**Authors:** Xiao-Qian Li, Lei Cheng, Tian-Fen Chen, Yi-Nuo Ma, Xiao-Hui Li, Ting-Yu Fu, Jing Xiao, Zhan-Zheng Zhao

**Affiliations:** ^a^ Nephrology, The First Affiliated Hospital of Zhengzhou University, Zhengzhou University, Zhengzhou, China; ^b^ College of Medicine, Zhengzhou University, Zhengzhou, China; ^c^ Laboratory Animal Platform of Academy of Medical Sciences, Zhengzhou University, Zhengzhou, China

**Keywords:** Circadian rhythm, chronic kidney disease, inflammation, energy metabolism, time-series RNA-seq

## Abstract

**Background:**

Chronic kidney disease (CKD) is driven by inflammation, fibrosis, and metabolic dysfunction. While circadian rhythm dysregulation is well documented in chronic disorders, its specific impact on CKD pathogenesis remains elusive.

**Methods:**

We performed four-hour interval time-series RNA sequencing on renal tissues from control and CKD mice. We used the JTK_CYCLE algorithm to identify rhythmic genes and categorize them as lost, acquired, or sustained in CKD; we subsequently performed focused bioinformatic analyses.

**Results:**

The renal circadian profile was substantially altered; acquired rhythmicity emerged as the dominant pattern, and core clock gene expression was disrupted. Kyoto Encyclopedia of Genes and Genomes (KEGG) analysis revealed that upregulated acquired-rhythmic genes in CKD were enriched in immune-inflammatory pathways; the expression of these genes peaked at Zeitgeber time (ZT) 12–16, consistent with a higher level of renal macrophage infiltration at ZT16 than at ZT0. Conversely, genes associated with nutrient and energy metabolism pathways were downregulated but acquired rhythmicity in CKD. Dapagliflozin improved renal function and restored the circadian expression rhythms of NR1D1 and p-BMAL1.

**Conclusions:**

CKD profoundly remodels the renal circadian transcriptome, driving immune-inflammatory and metabolic pathways into maladaptive rhythmicity. Furthermore, dapagliflozin can partially restore the expression of renal core clock genes.

## Introduction

Chronic kidney disease (CKD), the common end-stage of diverse kidney diseases, represents a global public health concern. Although inflammation [1], fibrosis [2], and metabolic dysfunction [3] are known to contribute substantially to the pathogenesis of CKD, the underlying mechanisms remain incompletely understood.

The circadian rhythm is an intrinsic oscillating system with an approximately 24-h cycle, orchestrating molecular functions and biological processes to facilitate adaptation to external changes. The circadian clock arises from a self-regulating feedback loop involving interconnected transcription factors encoded by core clock genes. The BMAL1 and CLOCK heterodimer constitutes the core of the circadian system, driving the transcription of downstream clock genes, such as *Pers*, *Crys*, *Nr1d1*, *Nr1d2*, and *Rors*. Elevated levels of PER and CRY protein complexes inhibit BMAL1–CLOCK activity, constituting the primary negative feedback loop. In addition, ROR proteins activate the expression of *Bmal1* and *Clock* to maintain circadian balance, counteracting the repressive effects of *NR1D1* and *NR1D2* in the secondary feedback loop [4].

The circadian rhythm is essential for the kidneys, governing numerous physiological processes, including the glomerular filtration rate, renal plasma flow, water and solute excretion, hormone signaling, and metabolism [5]. Clinical studies have identified a link between circadian rhythm disorders – such as a non-dipping blood pressure phenotype, disrupted sleep rhythms, or the presence of circadian syndrome – and the progression of CKD [6–8]. In animal models, alterations in external circadian cues, such as light/dark cycle reversal [9] and time-restricted feeding [10], have been shown to either exacerbate or reverse CKD progression, respectively. Furthermore, the critical role of core clock genes in kidney diseases is attracting attention, with most studies focusing on *Bmal1* and *Clock*. Deficiency in either *Bmal1* or *Clock* exacerbates fibrosis, elevates inflammation, and disrupts metabolic homeostasis in CKD and diabetic nephropathy [11–13]. Additionally, the cell-specific effects of the circadian clock in various renal compartments have been elucidated. Beyond its documented actions in the renal tubules, podocyte-specific *Clock* disruption triggers autophagy and increases albuminuria [14]. However, circadian alterations in the expression of other renal clock genes – particularly *Crys*, *Pers*, *Nr1d1*, *Nr1d2*, and *Rors*, which are integral components of the feedback loop – remain unclear. Moreover, the effects of circadian disruption on renal pathophysiology in CKD warrant further investigation. Therefore, we conducted time-series transcriptomic sequencing on the renal tissues of CKD mice, followed by a comprehensive bioinformatic analysis.

Sodium-glucose co-transporter 2 (SGLT2) mediates sodium and glucose reabsorption in the proximal tubules. Clinical and experimental investigations have firmly established SGLT2 inhibitors as an effective therapeutic strategy for CKD, largely owing to their pleiotropic effects and mechanisms of action [15]. Notably, Yoshioka et al. demonstrated that dapagliflozin administered to mice fed a high-fat diet at zeitgeber time 2 (ZT2) improved glucose metabolism more effectively than when administered at ZT14, highlighting the chronotherapeutic potential of dapagliflozin [16]. Therefore, we treated adenine-induced CKD mice with dapagliflozin to explore its potential impact on the renal core circadian clock.

## Methods

### Animal experiments

Eight-week-old male C57BL/6J mice (weighing approximately 20 g each) were obtained from SPF Biotechnology Co., Ltd. (Beijing, China). The mice were housed under standard laboratory conditions at the Laboratory Animal Center of Henan Province, with *ad libitum* access to food and water. The light/dark cycle was maintained from 8:00 AM to 8:00 PM, with 8:00 AM designated as ZT0. All experimental procedures were approved by the Animal Ethics Committee of the First Affiliated Hospital of Zhengzhou University (Approval No. ZZU-LAC20230825[07]). After a 1-week acclimatization period, the mice were randomly divided into two groups: (1) a control group (*n* = 18) fed an AIN93G diet (XT93G; Jiangsu Xietong Pharmaceutical Bio-engineering Co., Ltd., Nanjing, China) and (2) a CKD group (*n* = 18) fed the same diet supplemented with 0.25% adenine (A8626; Sigma-Aldrich, Shanghai, China) to induce CKD. Body weights were recorded weekly. After 4 weeks of feeding, the mice were anesthetized with tribromoethanol (M2920; Nanjing Aibei Biotechnology Co., Ltd., Nanjing, China) and euthanized via cervical dislocation. Kidneys were collected at six time points at 4-h intervals (ZT0, ZT4, ZT8, ZT12, ZT16, and ZT20). Three mice per group were euthanized at each time point.

For the dapagliflozin treatment experiment, mice were randomly allocated into three groups: (1) a control group administered 0.5% carboxymethylcellulose sodium via oral gavage (CTRL, *n* = 18); (2) a vehicle-treated CKD group administered 80 mg/kg adenine dissolved in 0.5% carboxymethylcellulose sodium (AD_Veh, *n* = 18); and (3) a dapagliflozin-treated CKD group administered 80 mg/kg adenine along with 10 mg/kg dapagliflozin dissolved in 0.5% carboxymethylcellulose sodium (AD_Dapa, *n* = 18). The remaining procedures were identical to those described above.

### Sample collection and measurements

One day prior to euthanasia, 24-h urine samples were collected using metabolic cages. Urinary albumin levels were determined using a Mouse Albumin ELISA Quantitation Kit (E99-134; Bethyl Laboratories, Montgomery, TX). Urinary creatinine levels were measured using the QuantiChrom Creatinine Assay Kit (DICT-500; BioAssay Systems, Hayward, CA). Urinary albumin levels were normalized to urinary creatinine levels. Blood samples were collected via retro-orbital venous plexus puncture, and serum was subsequently isolated. Serum creatinine levels were determined using the aforementioned QuantiChrom kit. Blood urea nitrogen levels were quantified using a commercial kit (C013-2-1; Nanjing Jiancheng Bioengineering Institute, Nanjing, China).

### Histological staining and immunohistochemistry

Immediately after euthanasia, one-quarter of the left kidney was fixed in 4% paraformaldehyde (BL539A; Biosharp, Hefei, China) for 24 h. The tissues were embedded in paraffin and sectioned at a thickness of 5 μm by Wuhan Servicebio Technology Co., Ltd. (Wuhan, China). The sections were stained with hematoxylin and eosin (H&E; G1120, Solarbio Science & Technology Co., Ltd., Beijing, China) and Sirius Red (G1472; Solarbio Science & Technology Co., Ltd., Beijing, China) according to the manufacturer’s instructions. Immunohistochemistry was performed using a primary antibody against F4/80 (GB113373; Wuhan Servicebio Technology Co., Ltd., Wuhan, China). Blocking reagents and secondary antibodies were applied according to the manufacturer’s protocol for the Rabbit Two-Step Method Test Kit (PV-9001; ZSJQ-Bio, Beijing, China). 3,3′-Diaminobenzidine (ZLI-9018, ZSJQ-Bio, Beijing, China) was used for signal detection, and hematoxylin (G1004; Servicebio, Wuhan, China) was used for counterstaining. For quantification, images of the peripheral renal tubular regions were captured at ×200 and ×400 magnification. The percentages of tubular dilation and interstitial collagen deposition were quantified using ImageJ-Fiji software (National Institutes of Health, Bethesda, MD).

### Transcriptome sequencing

For both the control and CKD groups, three biological replicates were prepared at each ZT. RNA extraction and transcriptome sequencing were conducted by OE Biotech Co., Ltd. (Shanghai, China). Total RNA was isolated using the mirVana miRNA Isolation Kit (catalog no. AM1561; Thermo Fisher Scientific, Waltham, MA). RNA integrity was assessed with an Agilent 2100 Bioanalyzer (Agilent Technologies, Santa Clara, CA), and only samples with an RNA Integrity Number ≥ 7 were included in subsequent analyses. RNA concentrations were determined using a NanoDrop 2000 spectrophotometer (Thermo Fisher Scientific, Waltham, MA). Libraries were constructed using the TruSeq Stranded mRNA LT Sample Prep Kit (Illumina, San Diego, CA) and subsequently sequenced on the Illumina platform. Raw sequencing data (raw reads) were processed using Trimmomatic; specifically, reads containing poly-N sequences and low-quality reads were filtered out to obtain clean reads. The fragments per kilobase million values for each gene were calculated using Cufflinks, and the read counts of individual genes were generated via HTSeq-count. The raw and normalized transcriptome data generated in this work were submitted to NCBI and can be accessed under the BioProject accession number PRJNA1354410.

### Rhythm analyses

Gene rhythmicity was analyzed using JTK_CYCLE (MetaCycle package in R) with Benjamini–Hochberg multiple testing correction. A *q*-value < 0.2 was used as the cutoff for rhythmicity. The parameters (mesor, amplitude, and acrophase) of rhythmic genes were determined through Cosinor analysis (https://cosinor.online/app/cosinor.php).

### Bioinformatic analyses

Differentially expressed genes (DEGs) were identified based on an absolute fold change > 2 and an adjusted *q*-value < 0.05. Gene Set Enrichment Analysis, Gene Ontology, and Kyoto Encyclopedia of Genes and Genomes (KEGG) enrichment analyses were performed using OECloud tools (https://cloud.oebiotech.com). Protein–protein interaction (PPI) analyses were conducted using the STRING database, and hub genes were screened with MCODE and CytoHubba in Cytoscape. The list of transcription factor–target genes was obtained from the Gene Transcription Regulation Database (http://gtrd.biouml.org/#!) via OECloud. Heatmaps were generated using the OECloud Platform (https://cloud.oebiotech.cn/#/home). The data were visualized as Venn diagrams, enrichment dot bubbles, fan diagrams, and ridgeline plots via the Wei Sheng Xin platform (https://www.bioinformatics.com.cn/). The mechanistic diagram was created using the Figdraw platform (https://www.figdraw.com/).

### Quantitative real-time PCR

RNA was extracted from kidney tissues using RNA Extraction Reagent (R401-1; Vazyme, Nanjing, China) according to the manufacturer’s protocols. RNA concentrations were determined with a NanoDrop spectrophotometer. Total RNA was reverse-transcribed into cDNA using RT mix with DNase (All-in-One) (R2020; US EVERBRIGHT, San Francisco, CA). Gene expression levels were quantified via quantitative real-time PCR using Hieff^®^ qPCR SYBR Green Master Mix (Low Rox Plus) (11202ES; Yeasen, Shanghai, China). Primers were obtained from Tsingke Biotechnology Co., Ltd. (Beijing, China). 18S rRNA was used as the internal reference.

### Western blotting

For protein extraction, renal tissues were homogenized in RIPA buffer (P0013B; Beyotime, Shanghai, China) supplemented with 1× General Protease Inhibitor Cocktail (EDTA-free) (GRF101; Epizyme Biotech, Shanghai, China) and 1× General Phosphatase Inhibitor Cocktail (100× stock solution) (GRF102; Epizyme Biotech, Shanghai, China). Total protein concentration was measured using a BCA Protein Assay Kit (P0010; Beyotime, Shanghai, China). The following primary antibodies were used: BMAL1 (14268-1-AP; Proteintech, Wuhan, China), NR1D1 (14506-1-AP; Proteintech, Wuhan, China), and GAPDH (10494-1-AP; Proteintech, Wuhan, China). Band intensities were quantified with ImageJ (Bethesda, MD) and normalized to GAPDH.

### Statistical analyses

Differences between two groups of quantitative data were analyzed using an unpaired Student’s *t*-test. Comparisons of fragments per kilobase million across ZTs were performed using a two-way analysis of variance followed by a Bonferroni post hoc correction. Data are displayed as the mean ± standard error of the mean (standard error of the mean). A *p* value < 0.05 was considered statistically significant. Data analysis and visualization were performed using GraphPad Prism software version 9 (GraphPad Software Inc., San Diego, CA).

## Results

### The circadian rhythm profile is reprogrammed in CKD mice

**Figure 1.**
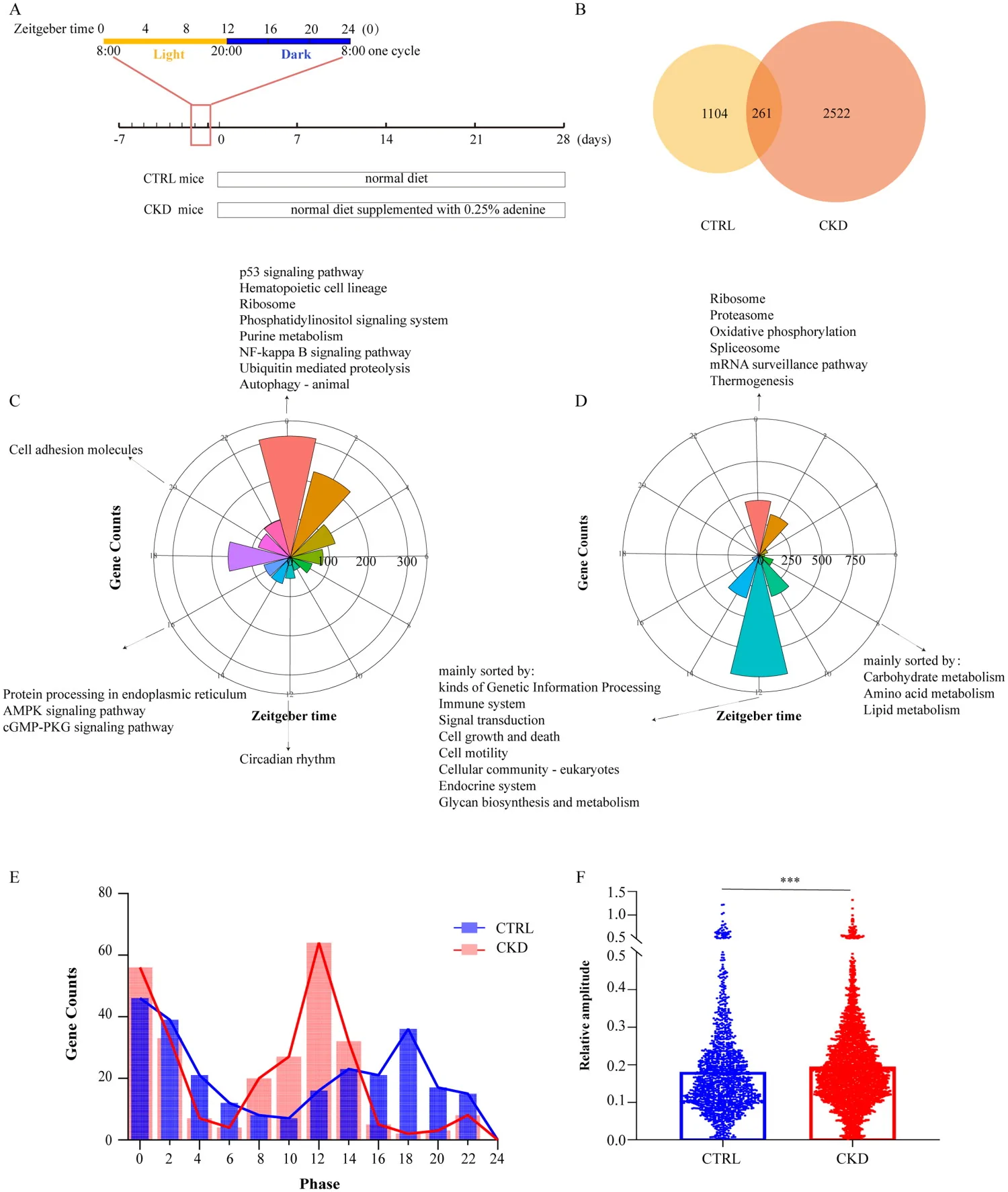
The circadian rhythm profile is reprogrammed in CKD mice. (A) Overview of the animal experimental design (*n* = 18 per group). (B) Venn diagram exhibiting the overlap of rhythmic genes identified by the JTK_CYCLE algorithm in control and chronic kidney disease (CKD) mice. (C) Rose plot of the phase distribution of rhythmic genes in control mice. The outer ring lists representative Kyoto Encyclopedia of Genes and Genomes (KEGG) pathways enriched among genes peaking at zeitgeber time (ZT) 0, ZT4, ZT8, ZT12, ZT16, and ZT20. (D) Rose plot of the phase distribution of rhythmic genes in CKD mice. The outer ring lists representative KEGG pathways enriched among genes peaking at ZT0, ZT4, ZT8, ZT12, ZT16, and ZT20. (E) Histogram comparing the phase distributions of genes retaining rhythmicity in control and CKD mice. (F) Relative amplitudes of the 261 genes that remained rhythmic in control and CKD mice. Data are presented as the mean ± standard error of the mean (SEM). A *p* value < 0.05 was considered statistically significant. ***p < 0.001.

The experimental scheme is illustrated in Figure 1(A). The mice with CKD exhibited decreased body weight, increased blood urea nitrogen and serum creatinine levels, an elevated urinary albumin-to-creatinine ratio, enlarged tubules, and severe renal interstitial fibrosis (Supplementary Figure 1A–E). These results confirmed the successful establishment of the CKD model. Subsequently, we performed time-series transcriptomic sequencing on the renal tissues and used JTK_CYCLE analysis to identify rhythmic genes. In total, 20,116 genes were detected in the control group, while 21,479 genes were detected in the CKD group. A total of 19,329 genes common to both groups were retained for downstream analysis. Specifically, 1,365 genes exhibited rhythmic expression in the control group, while 2,783 genes displayed rhythmicity in the CKD group; 261 genes overlapped between the two groups (Figure 1(B)). To determine the phase distribution and associated biological processes of rhythmic genes at each time point in the control and CKD mice, we generated rose plots and performed KEGG enrichment analyses. For the KEGG enrichment analysis, we grouped genes that peaked at ZT0 and ZT2 into the ZT0 category, and genes that peaked at ZT4 and ZT6 into the ZT4 category, applying this 4-h binning strategy across all time points. In the control group, the rhythmic genes primarily peaked around ZT0. Pathways such as the p53 signaling pathway and ribosome biogenesis were significantly enriched around ZT0 (the resting phase) (Figure 1(C)). In the CKD group, the rhythmic genes primarily peaked around ZT12 (the shift from the resting to the active phase). Metabolic pathways were predominantly enriched at ZT8, while pathways associated with cell growth and death, and the immune system, were enriched at ZT12 (Figure 1(D)). Regarding the 261 genes oscillating in both conditions, the phase distribution varied between the groups (Figure 1(E)), and the relative amplitude of these rhythmic genes was higher in the CKD group than in the control group (Figure 1(F)).

### Renal circadian clock gene expression is disrupted in CKD mice

**Figure 2.**
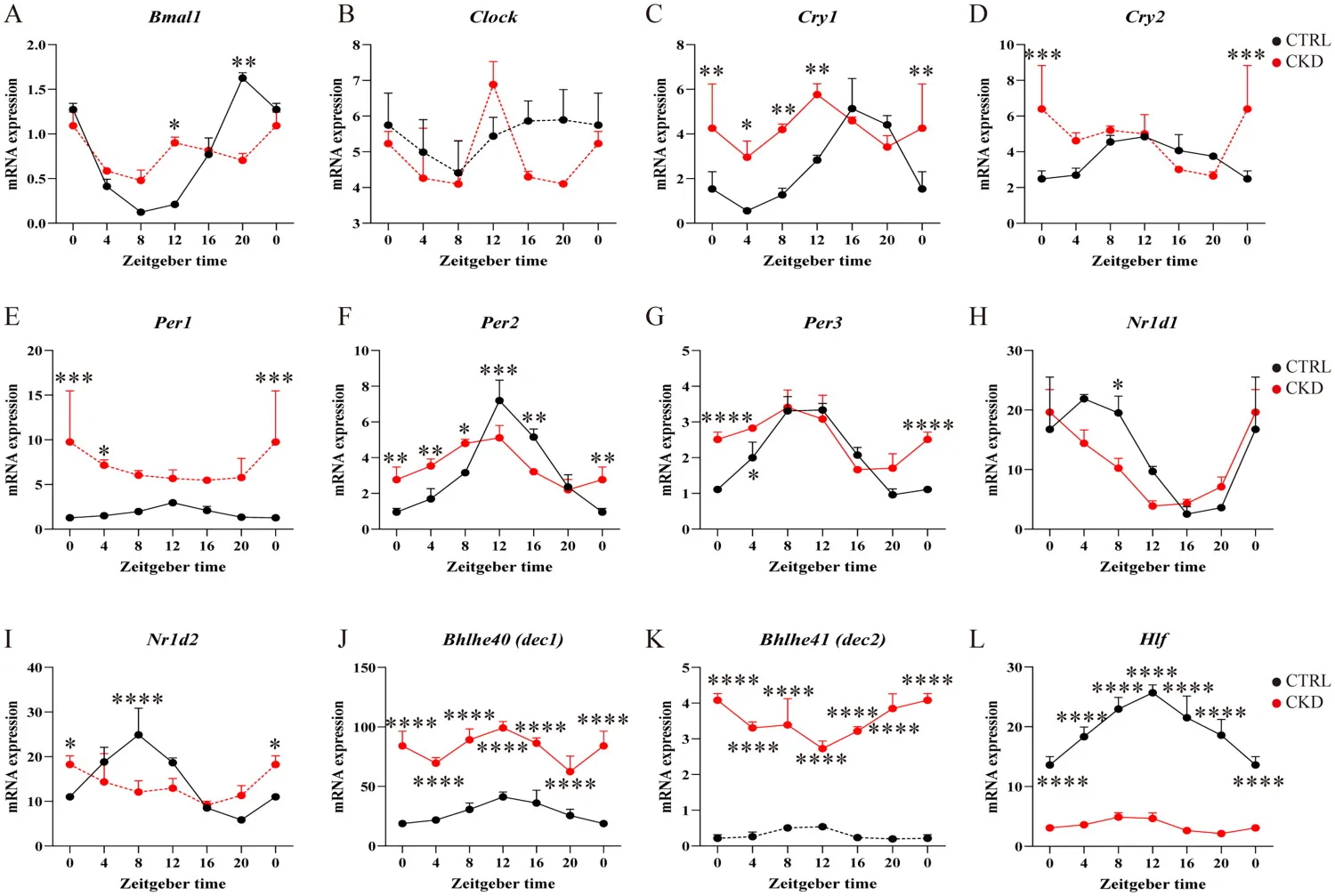
The circadian patterns of renal core clock genes in CKD mice. Renal circadian expression patterns of core clock genes, including (A) *Bmal1*, (B) *Clock*, (C) *Cry1*, (D) *Cry2*, (E) *Per1*, (F) *Per2*, (G) *Per3*, (H) *Nr1d1*, (I) *Nr1d2*, (J) *Bhlhe40* (*Dec1*), (K) *Bhlhe41* (*Dec2*), and (L) *Hlf*, in control (black lines) and chronic kidney disease (CKD) (red lines) mice over a 24-hour period. Solid lines denote rhythmic oscillation, whereas dotted lines represent arrhythmic expression. *n* = 3 per zeitgeber time (ZT) point in each group. Data are presented as the mean ± standard error of the mean (SEM). A two-way analysis of variance (ANOVA) with a Bonferroni post hoc correction was performed, and a *p* value < 0.05 was considered statistically significant. *p < 0.05; **p < 0.01; ***p < 0.001; ****p < 0.0001.

Because the renal circadian profile is regulated by core clock genes, we analyzed the circadian expression of the renal core clock components. When expression levels across the six ZTs were pooled for comparison between CKD and control mice, *Cry1*, *Per1*, *Bhlhe40*, and *Bhlhe41* were significantly upregulated in CKD, whereas *Hlf* expression was downregulated (Supplementary Figure 2). The circadian expression of these core clock genes was markedly altered in CKD. As illustrated in Figure 2, many core clock genes, such as *Bmal1*, *Cry2*, *Per1*, and *Nr1d2*, were rhythmic in the control mice, whereas their circadian rhythmicity was disrupted in the mice with CKD. Conversely, *Bhlhe41* became rhythmic only in the CKD group. We found that *Cry1*, *Per2*, *Per3*, *Nr1d1*, *Bhlhe40*, and *Hlf* were rhythmic in both control mice and mice with CKD. However, their rhythmic parameters were substantially altered in CKD, as determined by Cosinor analysis (Supplementary Table 1). Specifically, the mesors of *Cry1*, *Per3*, *Bhlhe40*, and *Hlf* differed, and the acrophases of *Per2*, *Per3*, *Nr1d1*, and *Hlf* were advanced in CKD. The relative amplitudes of *Per2*, *Nr1d1*, and *Hlf* were reduced in CKD.

### Biological functions of genes with disturbed rhythmicity in CKD

**Figure 3.**
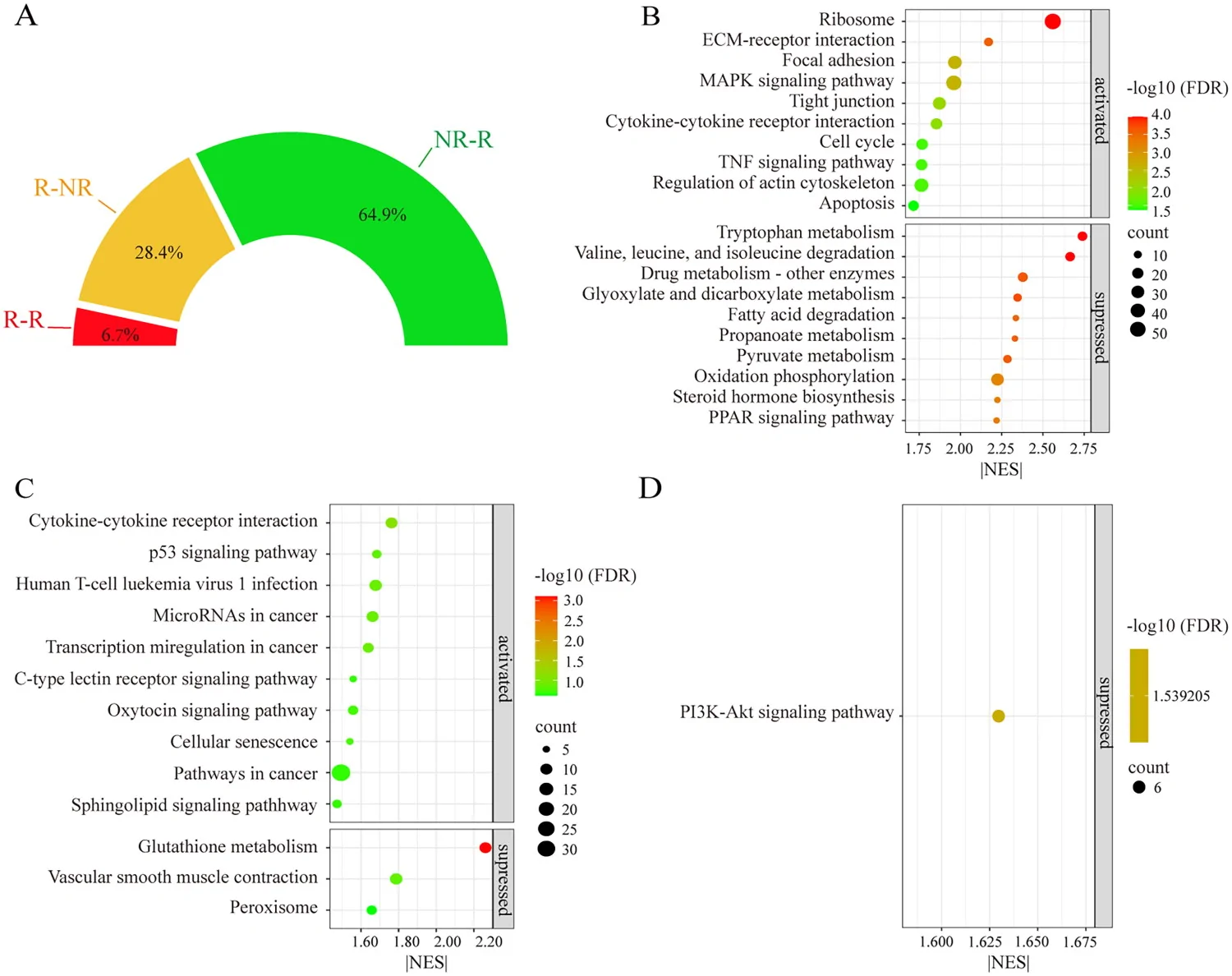
Biological functions of genes with disturbed rhythmicity in CKD. (A) Proportions of non-rhythmic to rhythmic (NR-R), rhythmic to non-rhythmic (R-NR), and rhythmic to rhythmic (R-R) genes in control versus chronic kidney disease (CKD) mice. Bubble plots displaying the top enriched biological processes determined via Gene Set Enrichment Analysis (GSEA) of genes in the (B) NR-R, (C) R-NR, and (D) R-R groups. Significance thresholds were set at a *p* value < 0.05, a false discovery rate (FDR) > 0.25, and an absolute normalized enrichment score (|NES|) > 1.5.

To better elucidate alterations of the circadian rhythm system in CKD, we categorized genes into four types based on their rhythmic patterns in control and CKD mice: non-rhythmic in both groups (NR-NR), rhythmic only in control mice (R-NR), rhythmic only in CKD (NR-R), and rhythmic in both conditions (R-R). Genes classified as NR-R, R-NR, and R-R were collectively designated as the circadian-dysregulated gene set (*n* = 3,887). The distribution of these genes was as follows: NR-R, 64.9% (*n* = 2,522); R-NR, 28.4% (*n* = 1,104); and R-R, 6.7% (*n* = 261), highlighting NR-R as the predominant type of circadian disruption in CKD (Figure 3(A)). Subsequently, Gene Set Enrichment Analysis was conducted on the different categories of circadian-dysregulated gene sets. For the NR-R genes, biological processes associated with inflammation, structural integrity, and the cell cycle were activated, whereas those involved in amino acid, lipid, and pyruvate metabolism were suppressed (Figure 3(B)). In the R-NR group, cytokine–cytokine receptor interactions and the p53 signaling pathway were highly activated, whereas glutathione metabolism was substantially suppressed (Figure 3(C)). Meanwhile, R-R genes were specifically enriched in the phosphatidylinositol 3-kinase-protein kinase B signaling pathway (PI3K–Akt) signaling pathway (Figure 3(D)).

### The circadian rhythm of immune- and inflammation-related pathways is dysregulated in CKD

**Figure 4.**
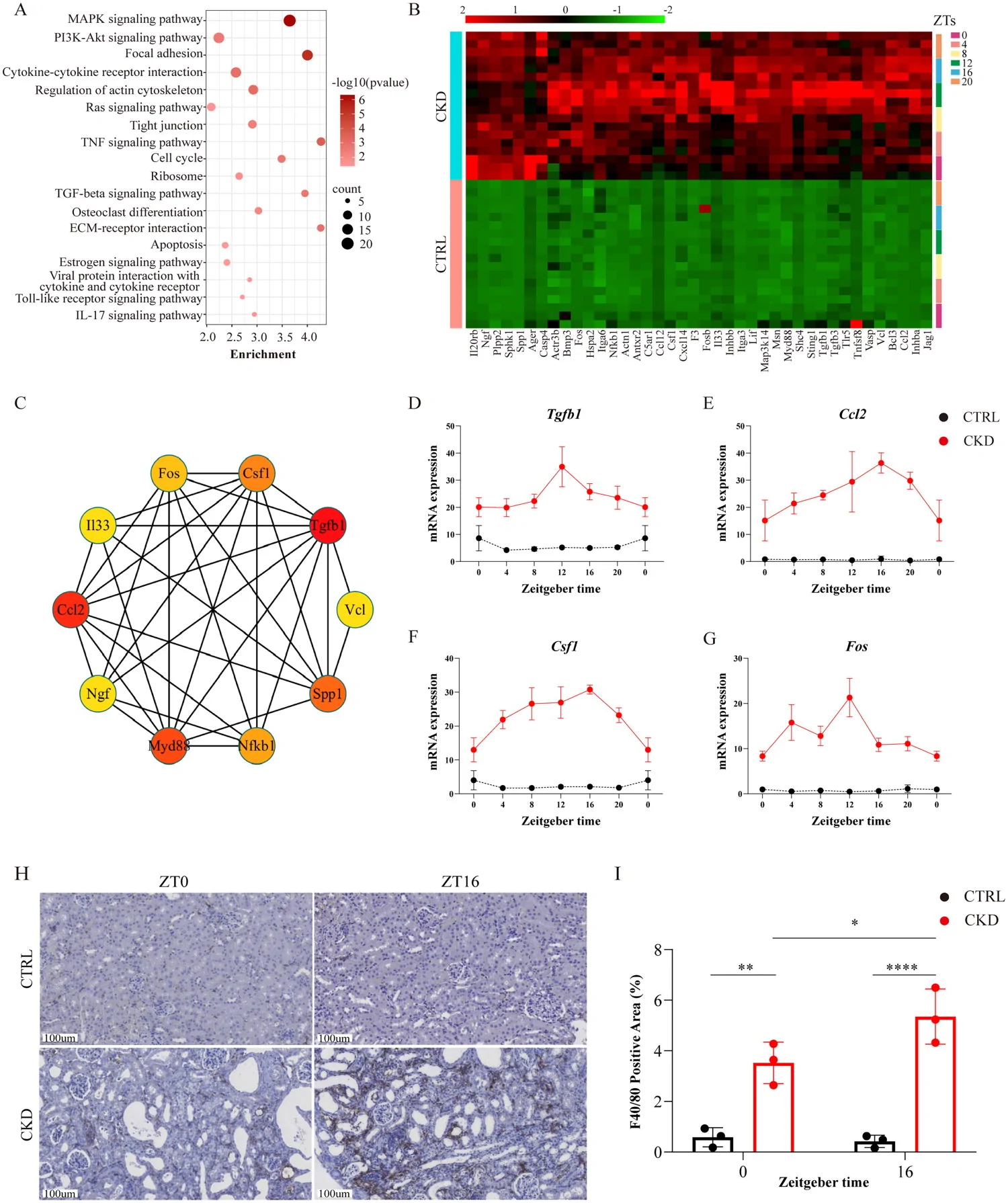
The circadian rhythms of immune- and inflammation-related pathways are dysregulated in CKD. (A) Kyoto Encyclopedia of Genes and Genomes (KEGG) bar plot showing pathways enriched among upregulated differentially expressed genes (DEGs) in the non-rhythmic to rhythmic (NR-R) group (*p* value < 0.05, Listhits ≥ 5, Human Diseases categories excluded). (B) Phase-ordered heatmap of the immune- and inflammation-related genes in control and chronic kidney disease (CKD) mice. (C) Top 10 hub genes identified via protein–protein interaction (PPI) analysis of immune- and inflammation-related genes. Color intensity is positively correlated with the CytoHubba degree. (D–G) Renal circadian expression patterns of *Tgfb1*, *Ccl2*, *Csf1*, and *Fos*. Three mice were included at each zeitgeber time (ZT) point per group. (H) Representative F4/80 immunohistochemical staining and (I) semi-quantitative analysis of kidney tissues at ZT0 and ZT16 in control and CKD mice (*n* = 3 per time point per group). Data are presented as the mean ± standard error of the mean (SEM). A *p* value < 0.05 was considered statistically significant. *p < 0.05; **p < 0.01; ****p < 0.0001.

Because NR-R represents the predominant pattern of circadian rhythm disruption in CKD, we focused on this subset. DEGs within the NR-R group were further screened to highlight circadian-dysregulated genes that might be implicated in the pathogenesis of CKD. KEGG enrichment analysis of upregulated DEGs in the NR-R group revealed several immune- and inflammation-related pathways, including the activated protein kinases signaling pathway, the PI3K–Akt signaling pathway, cytokine–cytokine receptor interactions, the tumor necrosis factor signaling pathway, viral protein interaction with cytokine and cytokine receptor, the Toll-like receptor signaling pathway, and the IL-17 signaling pathway (Figure 4(A)). These findings indicated that upregulated DEGs in the NR-R group were predominantly involved in immune and inflammatory responses. Subsequently, the immune- and inflammation-related genes were visualized in a heatmap. The expression phases of these genes clustered around ZT12–16 (Figure 4(B)). PPI analysis was conducted on the immune- and inflammation-related genes, identifying the top 10 hub genes, including *Tgfb1*, *Ccl2*, *Myd88*, *Spp1*, *Csf1*, *Nfkb1*, *Fos*, *Il33*, *Vcl* and *Ngf* (Figure 4(C)). The circadian expression patterns of *Tgfb1*, *Ccl2*, *Csf1*, and *Fos*, with peak expression around ZT12–16, are illustrated in Figure 4(D–G). Given that macrophages are major contributors to inflammatory responses in CKD, we performed F4/80 immunohistochemical staining on kidney tissues. Mice with CKD exhibited markedly higher levels of renal macrophage infiltration at ZT16 than at ZT0 (Figure 4(H,I)), a peak that aligns with the aforementioned transcripts. To exclude the confounding effects of nocturnal feeding habits, which might lead to increased nocturnal adenine intake, we administered adenine via gavage at ZT4. We found that the circadian expression patterns of these genes (*Bmal1*, *Cry1*, *Nr1d1*, *Tgfb1*, *Ccl2*, and *Csf1*) remained similar to those observed with *ad libitum* adenine feeding (Supplementary Figure 3).

### Key metabolism-related pathways acquire rhythm in CKD mice

**Figure 5.**
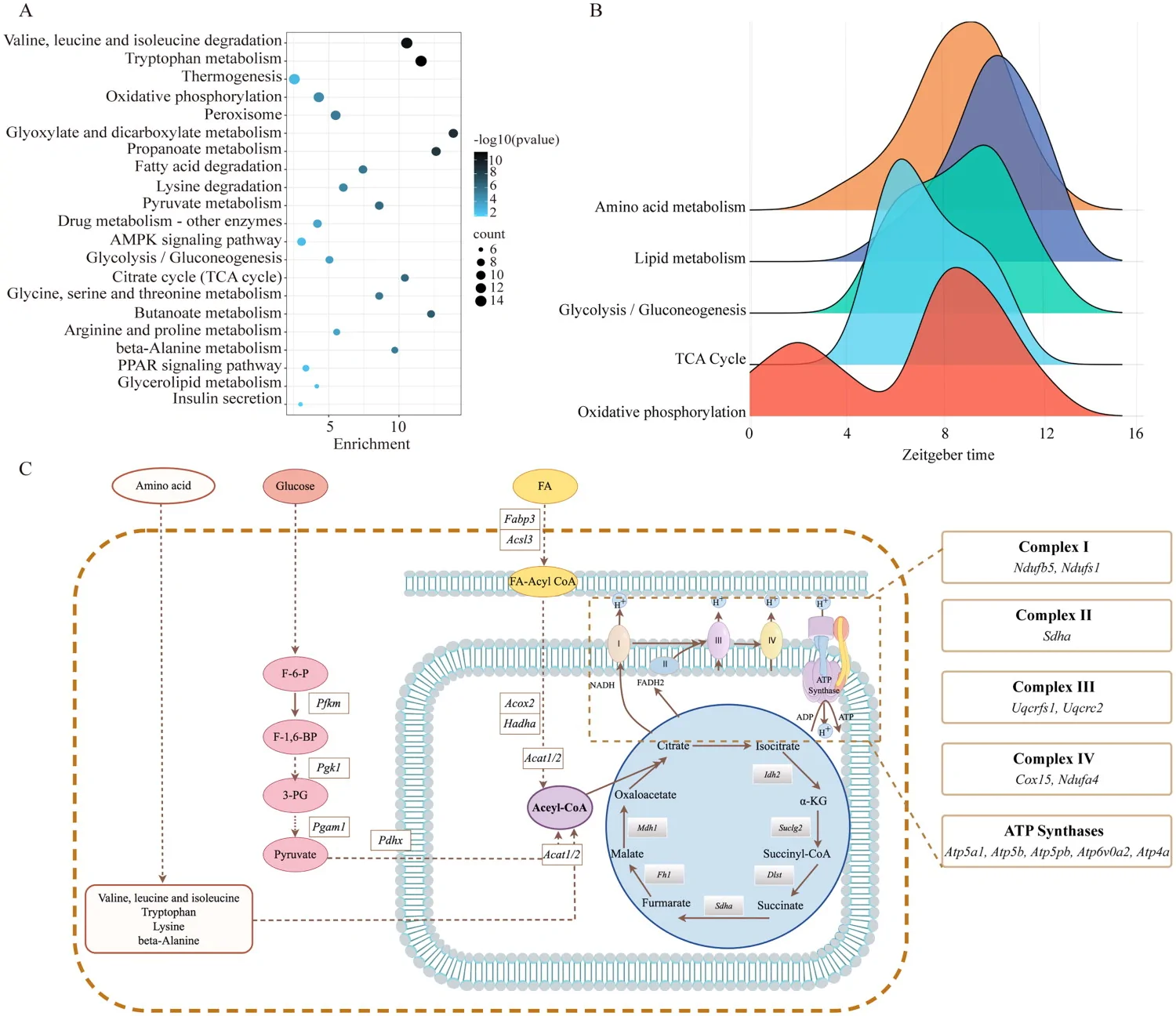
Key metabolism-related pathways acquire rhythmicity in CKD mice. (A) Kyoto Encyclopedia of Genes and Genomes (KEGG) bar plot showing all pathways enriched among downregulated differentially expressed genes (DEGs) in the non-rhythmic to rhythmic (NR-R) group (*p* value < 0.05 and Listhits ≥ 5, excluding Human Diseases categories). (B) Phase distribution of genes related to the metabolism of the three major nutrients (carbohydrates, lipids, and amino acids) and energy metabolism (tricarboxylic acid (TCA) cycle and oxidative phosphorylation) identified in the KEGG analysis. (C) Integrated metabolic pathway diagram highlighting the downregulated DEGs in the NR-R group.

KEGG analysis of downregulated DEGs in the NR-R group revealed substantial enrichment in the metabolism of the three major nutrients (carbohydrates, lipids, and amino acids) as well as in energy metabolism pathways (such as the tricarboxylic acid (TCA) cycle and oxidative phosphorylation) (Figure 5(A)). The phase distribution of the genes in these pathways spanned ZT0–ZT14, with a pronounced peak at ZT8–ZT10 (Figure 5(B)). A diagram of metabolic pathway integration was constructed to visualize the CKD-induced perturbations (Figure 5(C)). Genes involved in amino acid metabolism – including valine, leucine, isoleucine, tryptophan, lysine, and beta-alanine metabolism – were downregulated and acquired rhythmicity in mice with CKD. The expression levels of critical glycolytic genes, including *Pfkm*, *Pgk1*, and *Pgam1*, were downregulated, accompanied by reduced expression of *Pdhx*, a key component of pyruvate dehydrogenase kinase, which limits pyruvate entry into the TCA cycle. Concurrently, the expression of genes encoding key components involved in fatty acid transport and degradation/oxidation pathways, such as *Fabp3*, *Acsl3*, *Acox2*, *Acat1*, *Acat2*, and *Hadha*, was substantially perturbed. Within the TCA cycle, *Idh2*, *Suclg2*, *Dlst*, *Sdha*, *Fh1*, and *Mdh1* were coordinately repressed and became rhythmically expressed. Finally, the expression of genes encoding essential subunits of the respiratory chain complexes and adenosine triphosphate (ATP) synthase machinery was likewise influenced. Notably, the top 10 hub genes identified in the PPI analysis of downregulated DEGs in the NR-R group encoded essential components of the respiratory chain complexes and ATP synthase machinery (Supplementary Figure 4).

### A substantial proportion of circadian-dysregulated genes in CKD mice are direct transcriptional targets of core clock genes

To further reveal the link between core clock genes and the CKD-associated circadian-dysregulated genes, we extracted transcription factor–target gene pairs of core clock genes from the Gene Transcription Regulation Database and intersected them with the aforementioned immune- and inflammation-related DEGs and metabolism-related DEGs in the NR-R group. As shown in Table 1, the targets of NR1D1 showed the greatest overlap with our gene set, followed by those of BMAL1 and CLOCK. Subsequently, the immune- and inflammation-related DEGs and metabolism-related DEGs in the circadian-dysregulated gene sets (NR-R, R-NR, and R-R) were similarly extracted and intersected with this target gene set. Consistently, NR1D1, BMAL1, and CLOCK successively contributed the greatest number of target genes.

**Table 1.** The number of immune-inflammatory and metabolism-related genes with circadian rhythm disorder in CKD regulated transcriptionally by core clock genes.

TFs	The amount of genes regulating inflammation and metabolism	
	NR-R	NR-R, R-NR, R-R
NR1D1	148	216
BMAL1	123	191
CLOCK	108	163
NR1D2	103	152
BHLHE40	103	145
BHLHE41	31	43
RORa	66	99
RORc	80	118
NFIL3	31	52
HLF	1	3

### Dapagliflozin restored the aberrant expression of renal clock genes in CKD mice

**Figure 6.**
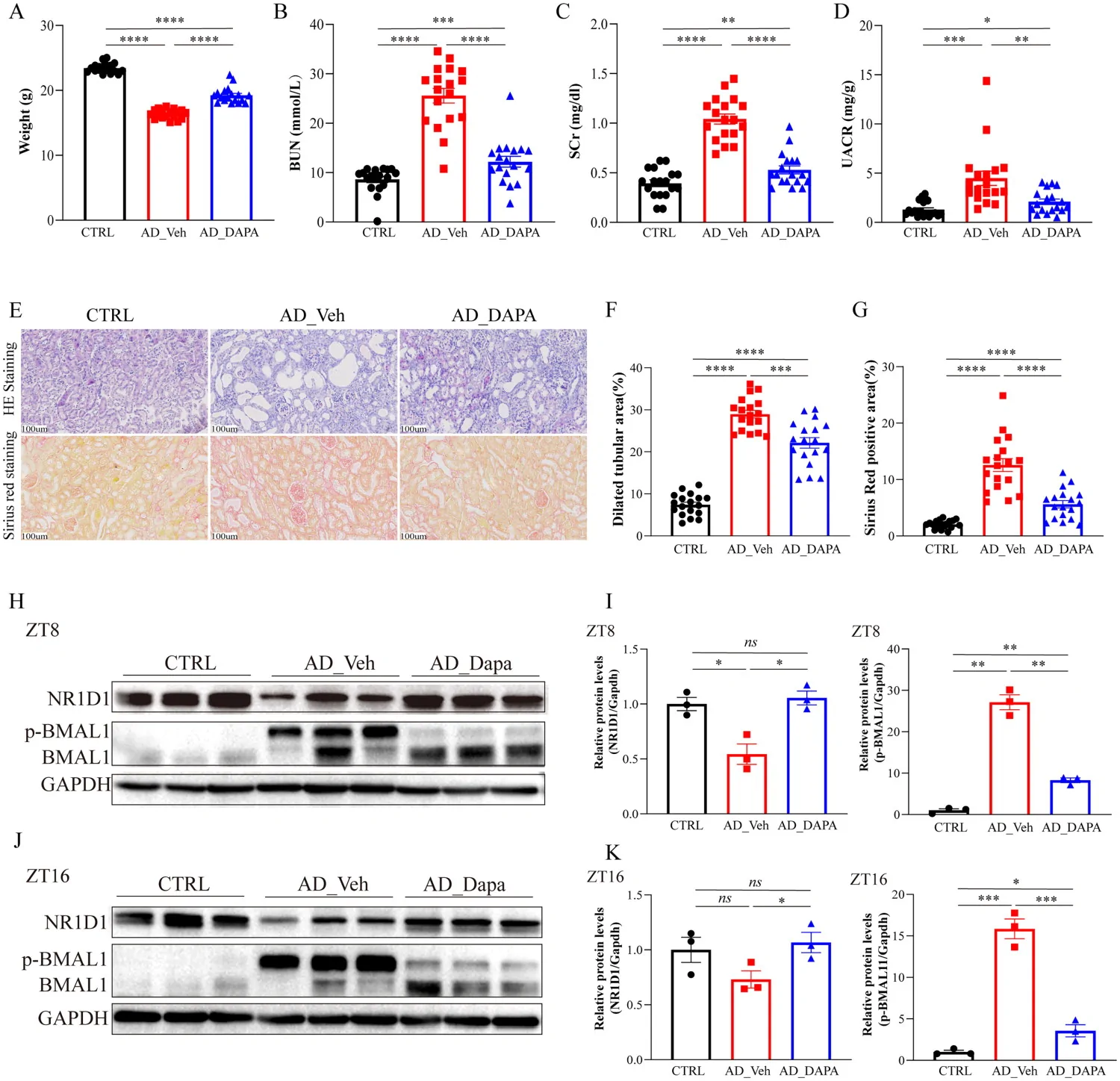
Dapagliflozin restored the aberrant expression of renal clock genes in CKD mice. Comparisons of (A) body weight, (B) blood urea nitrogen (BUN), (C) serum creatinine (SCr), and (D) the urinary albumin-to-creatinine ratio (UACR) among control, chronic kidney disease (CKD), and dapagliflozin-treated CKD mice. (E) Representative images of renal hematoxylin and eosin (H&E) and Sirius Red staining in the three groups of mice. (F, G) Semi-quantitative analyses of the dilated tubular area and Sirius Red-positive area in the three groups of mice. (H, I) Immunoblotting bands and semi-quantitative analyses of NR1D1 and p-BMAL1 in the three groups at zeitgeber time (ZT) 8. (J, K) Immunoblotting bands and semi-quantitative analyses of NR1D1 and p-BMAL1 in the three groups at ZT16. Each group consisted of 18 mice, and three mice were included per ZT point. Data are presented as the mean ± standard error of the mean (SEM). A *p* value < 0.05 was considered statistically significant. *p < 0.05; **p < 0.01; ***p < 0.001; ****p < 0.0001.

Next, we investigated whether dapagliflozin could influence renal core clock genes. Compared with untreated mice with CKD, those treated with dapagliflozin exhibited increased weight, decreased blood urea nitrogen and serum creatinine levels, reduced albuminuria, and alleviated renal tubular dilation and fibrosis (Figure 6(A–G)). Based on the findings in Table 1, we focused on evaluating the effects of dapagliflozin on NR1D1 and BMAL1. Renal tissues from ZT8 and ZT16 were selected for comparison considering the rhythmic patterns of these genes. At ZT8 and ZT16, no significant difference in BMAL1 levels emerged between mice with CKD and control mice. However, at ZT8, mice with CKD exhibited significantly elevated p-BMAL1 levels and reduced NR1D1 levels. Remarkably, dapagliflozin treatment in mice with CKD normalized these aberrant changes, restoring p-BMAL1 and NR1D1 levels toward physiological baseline (Figure 6(H,I), Supplementary Figure 5A). At ZT16, similar effects of dapagliflozin on p-BMAL1 and NR1D1 were observed in mice with CKD (Figure 6(J,K), Supplementary Figure 5B).

## Discussion

Using time-series transcriptome sequencing and JTK_CYCLE analysis, we detected a general reprogramming of renal circadian rhythms in CKD. Core clock genes are essential regulators of the renal circadian rhythm. To date, several pioneering studies have revealed that the dysregulation of renal core clock genes contributes to both tubular and glomerular injury in CKD. We found that, in addition to *Bmal1* and *Clock*, several other core clock genes, including *Cry2*, *Per1*, and *Nr1d2*, lost rhythmicity in CKD. *Bhlhe41* gained an oscillating pattern in mice with CKD. Meanwhile, genes retaining rhythmicity in both groups (*Cry1*, *Per2*, *Per3*, *Nr1d1*, *Bhlhe40*, and *Hlf*) exhibited changes in mesor, phase, or amplitude in CKD. Recent investigations have primarily focused on the roles of *Bmal1* and *Clock* [10–12], while fewer studies have investigated the other core clock genes in the kidneys. *Per1* can influence the circadian rhythm of the immune system in autoimmune diseases [17]. *Cry1* exerts circadian glycemic control by inhibiting gluconeogenesis in the liver [18,19]. *Bhlhe40* acts as a downstream effector of smad family proteins, inversely resetting the circadian clock [20,21]. *Nr1d1* suppresses inflammation and transactivates lipid-metabolism genes [22–24]. Our findings highlight the possible significance of renal core clock genes other than *Bmal1* and *Clock* in the fibrosis, inflammation, and metabolic dysfunction observed in CKD.

Existing studies have shown that the immune system and inflammation are regulated by circadian rhythms under certain conditions [25,26]. However, the relationship between immune and inflammatory responses and the circadian system in CKD remains unknown. Our study revealed that upregulated DEGs acquiring rhythmicity in CKD were primarily enriched in immune- and inflammation-associated pathways, with phases primarily distributed around ZT12–16. Of particular interest, we observed that the renal expression of *Ccl2* and *Csf1*, as well as macrophage infiltration (as manifested by F4/80 staining), increased and gained rhythmicity in CKD. This suggests that immune and inflammatory responses, such as macrophage function, are subject to dysregulated circadian control in CKD. Multiple immune cell types, such as monocytes, macrophages, mast cells, and neutrophils, have been found to possess intrinsic clocks [27]. These internal timers drive the temporal gating of various immune functions, including host–pathogen interactions, leukocyte trafficking, and the activation of innate and adaptive immunity [28]. Substantial evidence points toward clock gene disruption acting as a mediator of immune and inflammatory responses in chronic inflammatory diseases, such as rheumatoid arthritis [29] and asthma [30]. In macrophages, the NR1D1 and NR1D2 proteins represent a critical nodal point between the clock and immunity. For instance, NR1D1 and NR1D2 regulate the expression of a large number of inflammatory genes, including *Mmp9*, *Cx3cr1*, *Ccl2*, *Il1b*, and *Nlrp3/p65*, by binding to motifs in their enhancers or promoters [22,23]. This allows them to modulate macrophage inflammatory functions, including adhesion, migration, and activation. Furthermore, the disruption of *Bmal1*, *Clock*, *Crys*, or *Pers* also affects the inflammatory immune response in macrophages [31]. Further studies are warranted to clarify the mechanisms underlying the circadian regulation of immune and inflammatory responses in CKD.

Our subsequent analysis revealed that a subset of genes involved in metabolism-related pathways – especially those associated with mitochondrial respiration, including the TCA cycle and oxidative phosphorylation – were downregulated and acquired rhythmicity in CKD. This implies that the circadian regulation of mitochondrial metabolism might be disrupted in CKD, which could play a significant role in its pathogenesis. Mitochondria exhibit high structural and functional dynamism, and diminished flexibility in this regard correlates with metabolic disorders. Emerging evidence has indicated that multiple mitochondrial functions, including biogenesis, mitophagy, dynamics, and respiration, are regulated by the circadian system [32]. Regarding mitochondrial respiration, several mechanisms have been shown to control its circadian rhythm. For instance, the selective knockdown of *Bmal1* in the liver resulted in a reduced oxygen consumption rate when cells were exposed to a glycolytic substrate [33]. Additionally, the circadian rhythm of dynamin-related protein 1 mediates circadian oscillations of mitochondrial fission-fusion dynamics, oxidative phosphorylation, and ATP generation [34]. Several reports have shown that a dysfunctional circadian clock induces fragmented mitochondrial morphology, resulting in diminished mitochondrial respiration [35]. Furthermore, the circadian oscillation of the adenosine triphosphate/adenosine monophosphate ratio, which serves as a marker of mitochondrial respiration, can conversely influence core clock genes, such as *Pers* and *Crys*, via the adenosine 5’-monophosphate-activated protein kinase pathway [36]. Taken together, our analysis indicates that the circadian regulation of mitochondrial respiration is likely disrupted in CKD. Further experiments are needed to clarify the specific mechanisms involved.

SGLT2 inhibitors have emerged as one of the most notable advancements in nephrology in recent years. These agents offer substantial cardiorenal protection for patients with CKD, regardless of their diabetes status [37]. The mechanisms of action of SGLT2 inhibitors are highly complex. Wang et al. found that administering dapagliflozin to spontaneously hypertensive rats without heart failure upregulated the expression levels of *Per3*, *Bhlhe41*, and *Nr1d1* [38]. Glucose metabolism efficiency was improved in mice fed a high-fat diet and treated with dapagliflozin administered at ZT2 compared with mice administered the drug at ZT14 [16]. These investigations highlight a potential effect of dapagliflozin on the circadian rhythm; therefore, we explored whether dapagliflozin could affect the renal core clock in mice with CKD. We found that NR1D1 levels were decreased and p-BMAL1 levels were increased in mice with CKD, and these trends were substantially restored following dapagliflozin treatment. The ability of dapagliflozin to restore the balance of core clock components suggests that it might exert protective effects in normoglycemic CKD by targeting circadian rhythms. Small-molecule modulators, such as SR9009, are currently restricted to animal experiments due to their potential multisystem side effects [39]. Clinically, pharmacological interventions targeting the circadian clock primarily involve melatonin, which exerts its effects on the suprachiasmatic nucleus rather than on peripheral oscillators. Our results highlight the possibility of therapies targeting the circadian clock in peripheral oscillators.

Our study has several limitations. First, the kidney is composed of various cell types, each with distinct roles in pathophysiological states. Because our study focused on whole renal tissue, we could not elucidate diurnal rhythm variations within specific cellular components, such as renal tubular cells and podocytes. Second, our investigation was restricted to the transcriptomic level and did not further elucidate the impact of diurnal rhythm disturbances in CKD using metabolomic, lipidomic, or proteomic approaches. Third, the etiology of the adenine-induced CKD model differs from that of human CKD, in which diabetes and hypertension are the leading causes. Therefore, the generalizability of our findings must be verified in additional CKD models in future studies. In addition, dapagliflozin was administered concurrently with adenine in the present study, indicating that its effects on renal progression and circadian disruption were primarily preventive. Because clinical management focuses mainly on treating established disease, further investigations are warranted to evaluate the therapeutic efficacy of dapagliflozin in reversing circadian misalignment in established CKD. Finally, only male mice were included in this study. Given the known sex differences in circadian rhythms, the present findings may not be applicable to female mice [40].

## Conclusions

Our study revealed that the circadian rhythm profile is reprogrammed in CKD. The circadian rhythms of immune-inflammatory and metabolic processes were dysregulated in CKD. Moreover, the protective effects of dapagliflozin in CKD may be linked to the restoration of these circadian rhythms.

## Supplementary Material

Supplemental Material

Supplemental Material

Supplemental Material

Supplemental Material

Supplemental Material

Supplemental Material

## Data Availability

The data that support the findings of animal experiments are available from the corresponding author, Zhan-Zheng Zhao, upon reasonable request. The data that support the findings of this study are openly available in NCBI at https://www.ncbi.nlm.nih.gov/, reference number PRJNA1354410.
