# Supplementary material for: Circadian reprogramming of inflammation and metabolism in chronic kidney disease
Source: Ren Fail. 2026 Sep 6;48(1):2718935. doi: 10.1080/0886022X.2026.2718935 (PMC13552315; doi:10.1080/0886022X.2026.2718935)
Supplement: Supplementary file 1 [file IRNF_A_2718935_SM7961.pdf]

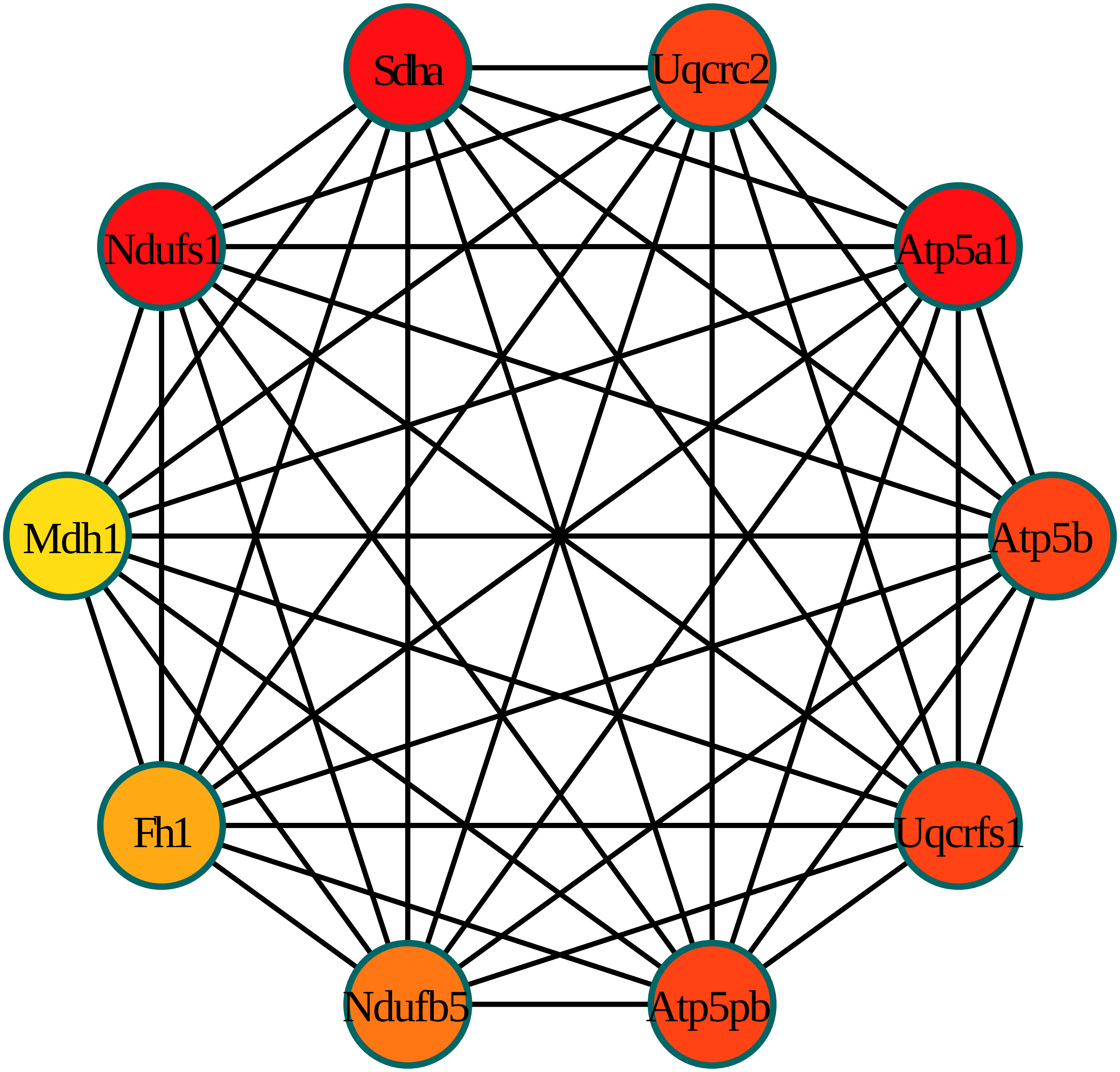
Sdha
Uqcrc2
Ndufs1
Atp5a1
Mdh1
Atp5b
Fh1
Uqcrfs1
Ndufb5
Atp5pb
